# Analysis of Tanner stage in children conceived after the diagnosis of infertility: the DESCRT study

**DOI:** 10.1007/s10815-025-03395-8

**Published:** 2025-02-07

**Authors:** Jane Y. Liu, Richard Hu, Robert H. Lustig, David Huang, Amanda J. Adeleye, Paolo Rinaudo, Marcelle I. Cedars, Lydia B. Zablotska

**Affiliations:** 1 School of Public Health, University of California Berkeley, Berkeley, CA 94720 USA; 2 Department of Epidemiology and Biostatistics, University of California, San Francisco, CA 94158 USA; 3 Department of Pediatrics, University of California, San Francisco, CA 94158 USA; 4 Department of Obstetrics Gynecology and Reproductive Sciences, University of California, San Francisco, CA 94158 USA; 5 Department of Obstetrics and Gynecology, University of Chicago, Chicago, IL 60637 USA

**Keywords:** ART, ICSI, IVF, Tanner stage, Puberty

## Abstract

**Purpose:**

Use of assisted reproductive technology (ART) could lead to abnormal pubertal development in children. We compared pubertal development in children conceived using ART to non-in vitro fertilization fertility treatment (NIFT) and unassisted (UA) conception.

**Methods:**

Children from the Developmental Epidemiological Study of Children through Reproductive Technology (DESCRT) were assessed for pubertal development according to the standardized protocol. Tanner staging (breast, testes, and pubic hair development) was compared between ART, NIFT, and UA-conceived children. Differences were analyzed using Wilcoxon rank-sum test, Pearson’s chi-squared test, and Fisher’s exact test. Multivariable logistic regression was used to assess association between method of conception and pubertal development.

**Results:**

The sample included 290 children (164 boys and 126 girls) at median age 6 years (range 4 to 18 years); 229 were conceived using ART compared to a combined reference group of 29 conceived via NIFT and 32 via UA. Statistical analyses adjusted for children’s age, height, and weight showed statistically non-significant trends wherein boys conceived via ART tended to be in later Tanner stages for pubarche (OR = 2.33, 95% CI 0.44–12.21) and gonadarche (OR = 4.27, 95% CI 0.44–41.99), and girls tended to be in later stages for pubarche (OR = 4.29, 95% CI 0.40–45.62) and thelarche (OR = 2.23, 95% CI 0.35–14.03) compared to the reference group.

**Conclusion:**

As ART becomes more widespread, developmental concerns become increasingly prominent. While results were statistically non-significant, we observed a trend toward differences in pubertal development between ART-conceived children and those conceived without assistance or NIFT.

## Introduction

Over the past several decades, the use of assisted reproductive technology (ART), including in vitro fertilization (IVF) and intracytoplasmic sperm injection (ICSI), to overcome infertility has increased in the USA, with approximately 2% of all infants born annually being conceived with ART [1]. Since the first infant born following ART-conception in 1978, many children born with the use of ART have now reached adulthood. While this treatment has successfully helped many individuals build families, it may be associated with potential long-term health risks for offspring during various stages of life, including cardiometabolic and pubertal outcomes during childhood and adolescence [2, 3].

The onset of puberty typically occurs in girls between 8 and 13 years and in boys between 9 and 14 years [4]. However, growing evidence indicates a shift to an earlier pubertal onset, especially in girls, due to a mix of both environmental and genetic factors [5]. Children experiencing early puberty are at risk for accelerated skeletal maturation, breast cancer [6], psychosocial difficulties, and high-risk behaviors, such as substance abuse [7, 8] and cardiovascular disease [9–11]. Reduced fetal growth [12], early life malnutrition [13], and childhood obesity are risk factors for early puberty [14–19]. Furthermore, environmental exposures, particularly to endocrine-disrupting chemicals, are another risk factor for early puberty [20–22].

While puberty is a key milestone in the transition from childhood to adulthood, pubertal development has seldom been studied in ART populations, with inconsistent findings [23–28]. Several studies in ART populations have concluded pubertal development is comparable between the IVF- or ICSI-conceived and children conceived without assistance, as indicated by Tanner staging [23–26]. In contrast, other studies reported trends toward earlier mean age of pubertal initiation in girls, and later mean age of pubertal initiation in boys conceived with ART [27]. The exact mechanism underlying this process remains unknown, but it is hypothesized epigenetic changes in the embryos or placenta may disturb future growth and predisposition individuals to disease [29, 30]. For example, epigenetic adaptation mechanisms in the placenta established during ART-associated pregnancy increases the risk of developing metabolic diseases later in life [31].

Developmental Epidemiological Study of Children through Reproductive Technology (DESCRT) is a large, multiethnic cohort assembled to determine what parental factors or specific ART may influence the risk for adverse cardiometabolic outcomes among children so conceived [32]. During in-person visits, children were also assessed for Tanner stage of puberty development. This study aimed to identify potential differences in the pubertal development of girls and boys by mode of conception, including ART (IVF or ICSI), non-IVF fertility treatment (NIFT; including ovulation induction with or without intrauterine insemination), and unassisted conception (UA). We hypothesized that children conceived with ART may commence puberty earlier, as measured by Tanner staging, compared to children conceived with NIFT or UA.

## Materials and methods

### Study population and participants

The DESCRT cohort is based on a patient population seen at the Center for Reproductive Health (CRH) at the University of California, San Francisco (UCSF). This cohort study has been previously described [32]. Briefly, this retrospective arm of the study was conducted during 2017–2022 and includes pregnancies and the resultant children born to parents who conceived after consultation for infertility or procreative management (e.g., fertility assistance for same-sex couples or preimplantation genetic testing for genetic conditions) at UCSF CRH starting in 2001. Infertility was defined as the inability to conceive after 12 months of regular unprotected intercourse (Zegers-Hochschild et al., 2017). Eligible children were conceived as a result of (1) unassisted (UA) conception to sub/infertile couples; (2) non-IVF fertility treatments (NIFT) such as ovulation induction (OI) or ovarian stimulation (with the use of letrozole, clomiphene citrate, or gonadotropins) with or without intrauterine insemination (IUI); or (3) ART, which included in vitro fertilization (IVF) or intracytoplasmic sperm injection (ICSI) followed by fresh or frozen embryo transfer (FET). Compared to several prior studies exploring the influence of ART on offspring health, most parents/couples in DESCRT were diagnosed with sub/infertility, which is recognized as an important factor to consider when investigating the specific effects of ART [33].

During 2017–2022, we approached all 7422 children who were born after a visit to the CRH during 2001–2017 at 24 weeks of gestation or greater. Of these, 1310 (18%) could be traced and agreed to participate in the study.

### Data collection

DESCRT is a cohort study that includes collection of patient clinical data from electronic medical records (EMRs), information on lifestyle and environmental factors from multiple questionnaires, offspring anthropometric and laboratory assessments from in-person visits, and biospecimens from both children and parents.

Children 4 years of age and older were gathered at the Pediatric Clinical Research Center for an in-person evaluation of their cardiometabolic health and outcomes. During this assessment, fasting blood and anthropomorphic measurements were collected. Developmental milestones assessed included Tanner staging, BMI, blood pressure, bioelectrical impedance analysis (BIA), measurements of fat composition including skinfold thickness, and waist circumference.

### Exposure and outcome

The primary exposure was the method of conception. We compared children conceived by ART (exposure group) to the combined group of children conceived either via UA or NIFT ovulation induction (reference group). The ART group was further subdivided for additional analyses into fresh and frozen embryo transfer.

Our primary outcome was Tanner stage at the age of offspring evaluation. Tanner staging was performed by experienced pediatric nurses according to a standardized protocol; the nurses were blinded to the mode of conception of the children during the in-person examination. Tanner stage is a rating from 1 to 5 of physical development as children progress through puberty [34, 35]. Tanner stage was evaluated for pubic hair in both boys and girls (pubarche), for testicular volume in boys (gonadarche), and for breast development in girls (thelarche). In our analysis, each of the Tanner stage outcomes of pubic hair, testicular volume, and breast development was dichotomized into two groups: Tanner stage 1 (prepubertal) vs. Tanner stages 2–5 (pubertal). Tanner stages have been dichotomized in other studies as both exposure [36–38] and outcome [39–42].

### Statistical analyses

We used mean ± SD to describe normally distributed continuous variables, and absolute frequencies and percentages for categorical data. Comparisons of reference and ART groups were conducted by Wilcoxon rank-sum test for continuous variables, Pearson’s chi-squared test for categorical variables, and Fisher’s exact test for sparsely distributed categorical data.

We used multivariable logistic regression with adjustment for potential confounders to estimate odds ratios (ORs) with 95% confidence intervals (CI) for the effect of method of conception on Tanner stage presentation. All analyses were conducted separately for boys and girls. Potential confounders were selected based on literature review and clinical knowledge, and included child age, child height, child weight, child waist circumference, average tricep skinfold thickness, parental education, household income at time of conception and at time of study, parental medical history, child race/ethnicity, maternal age at conception and delivery, child gestational age at delivery, child birthweight, and any maternal antepartum complications (e.g., hypertension, pre-gestational diabetes). In addition, we tested BMI-for-age, height-for-age, and weight-for-age *z*-scores calculated using reference tables from the Centers for Disease Control and Prevention but each case resulted in poor model prediction (results not shown).

To evaluate confounding, we examined three criteria: potential confounders are (1) independent risk factors for the outcome irrespective of the main exposure; (2) associated with the exposure of interest; and (3) not in the causal pathway between exposure and outcome. We used stepwise forward selection with a *p*-value of 0.05 and ability to improve model’s predictive accuracy, as assessed by pseudo-*R* squared method [43], to select confounders for our final model. Of note, we were unable to test socioeconomic factors such as parental education and household income due to a substantial proportion of respondents who declined to report these data (approximately one-third in both instances). Race but not ethnicity met the criteria for confounding described in the “Materials and methods” section; however, race did not noticeably improve predictive ability of the model for boys and led to overfitting (perfect prediction) in girls; thus, we did not include it in our final model. Waist circumference and mean tricep skinfold thickness both had moderate correlations with child weight (coefficient range 0.4–0.7) and were not included in the final model to avoid collinearity. Neither gestational age at delivery nor maternal age at conception was a confounder, nor did they appreciably improve model fit. We selected child age, child height, and child weight to be included as confounders in the final model. All analyses were performed using STATA software, version 18.0 [44].

## Results

A total of 309 children presented for in-person assessment of cardiometabolic outcomes (24% of the 1302 children enrolled in the DESCRT); of these, 19 children did not meet the age-related inclusion criteria or otherwise did not undergo baseline measurements, leaving 290 children (164 boys, 126 girls) for analyses (94% of those evaluated for Tanner stage).

### Descriptive characteristics

Table 1 presents the distribution of descriptive characteristics of the study sample. The overall median age of children was 6 years (range, 4 to 18 years). In addition, the median ages of boys and girls separately were both 6 years (boys’ interquartile range, 5 to 10 years; girls’ interquartile range, 4 to 10 years; *p* = 0.83).

**Table 1:** Descriptive characteristics of the study sample, by method of conception, DESCRT study (2017–2022)

Characteristic	Overall (*n* = 290)	UA + NIFT (*n* = 61)	ART (*n* = 229)	*p*-value^a^
Sex, *n* (%)				0.66
Girls	126 (43.5)	28 (45.9)	98 (42.8)	
Boys	164 (56.5)	33 (54.1)	131 (57.2)	
Age (years), mean (SD)	7.5 (3.5)	8.2 (3.5)	7.2 (3.5)	0.02
Female	7.3 (3.6)	8.4 (3.6)	6.9 (3.5)	0.02
Male	7.6 (3.5)	8.1 (3.5)	7.5 (3.5)	0.33
Missing, *n* (%)	0 (0)	0 (0)	0 (0)	
Ethnicity, *n* (%)				0.80
Hispanic	26 (9.0)	4 (6.5)	22 (9.6)	
Non-Hispanic	237 (81.7)	48 (78.7)	189 (82.5)	
Missing, *n* (%)	27 (9.3)	9 (14.8)	18 (7.9)	
Race, *n* (%)				0.37
White	168 (57.9)	30 (49.2)	138 (60.3)	
Asian or Pacific Islander	44 (15.2)	9 (14.8)	35 (15.3)	
Black or African American	5 (1.7)	0 (0)	5 (2.2)	
Multiple races	47 (16.2)	13 (21.3)	34 (14.9)	
Missing, *n* (%)	26 (9.0)	9 (14.7)	17 (7.4)	
Current household income, *n* (%)				0.38
Less than $50,000	2 (0.7)	0 (0)	2 (0.9)	
$50,000 to $74,999	4 (1.4)	2 (3.3)	2 (0.9)	
$75,000 to $99,999	6 (2.1)	2 (3.3)	4 (1.7)	
$100,000 to $149,999	28 (9.7)	7 (11.5)	21 (9.2)	
$150,000 to $199,999	23 (8.0)	3 (4.9)	20 (8.7)	
$200,000 or more	134 (46.2)	25 (41.0)	109 (47.6)	
Missing, *n* (%)	93 (32.1)	22 (36.1)	71 (31.0)	
Mother’s highest education, *n* (%)				> 0.9
Some high school, no diploma	1 (0.3)	0 (0)	1 (0.4)	
High school graduate or GED	1 (0.3)	0 (0)	1 (0.4)	
Some college credit, no degree	7 (2.4)	1 (1.6)	6 (2.6)	
Associate degree, trade/vocational training	2 (0.7)	0 (0)	2 (0.9)	
Bachelor’s degree	73 (25.2)	16 (26.2)	57 (24.9)	
Advanced degree	113 (39.0)	22 (36.1)	91 (39.7)	
Missing, *n* (%)	93 (32.1)	22 (36.1)	71 (31.0)	
Height (cm), mean (SD)	128 (21)	133 (22)	127 (21)	0.04
Female, ages 4–10	118 (14)	123 (14)	117 (13)	0.06
Female, ages 11–18	158 (14)	161 (11)	157 (15)	0.77
Male, ages 4–10	121 (14)	122 (15)	121 (14)	0.81
Male, ages 11–17	159 (12)	157 (13)	160 (12)	0.37
Weight (kg), mean (SD)	29 (14)	32 (15)	28 (14)	0.02
Female, ages 4–10	23 (8)	26 (10)	22 (7)	0.04
Female, ages 11–18	48 (13)	50 (19)	47 (11)	0.78
Male, ages 4–10	24 (8)	26 (10)	23 (7)	0.45
Male, ages 11–17	50 (13)	45 (10)	52 (14)	0.20
Tricep skinfold thickness (mm), mean (SD)	18 (9)	22 (11)	17 (8)	< 0.01
Missing, *n* (%)	20 (6.9)	3 (5.0)	17 (7.4)	
Birthweight (g), mean (SD)	3103 (568)	3235 (583)	3071 (562)	0.05
Missing, *n* (%)	22 (7.6)	10 (16.4)	12 (5.2)	
Maternal age at delivery (years), mean (SD)	38.1 (4.8)	36.2 (3.1)	38.6 (5.0)	< 0.001
Missing, *n* (%)	54 (18.6)	8 (13.1)	46 (20.1)	
Gestational age at delivery (days), mean (SD)	270 (15)	273 (15)	269 (15)	0.04
Missing, *n* (%)	16 (5.5)	6 (9.8)	10 (4.4)	
Born as part of multiple birth, *n* (%)				< 0.001
Yes	62 (21.4)	3 (4.9)	59 (25.8)	
No	220 (75.9)	55 (90.2)	165 (72.0)	
Missing, *n* (%)	8 (2.7)	3 (4.9)	5 (2.2)	
Birthweight (g) excluding multiple births, mean (SD)	3246 (523)	3256 (583)	3242 (506)	0.75
Missing, *n* (%)	13 (5.9)	7 (12.7)	6 (3.6)	
Maternal age at delivery (years) excluding multiple births, mean (SD)	38.2 (4.8)	36.3 (3.1)	38.9 (5.1)	< 0.01
Missing, *n* (%)	40 (18.2)	5 (9.1)	35 (21.2)	
Gestational age at delivery (days) excluding multiple births, mean (SD)	273 (15)	274 (15)	273 (15)	> 0.9
Missing, *n* (%)	7 (3.2)	2 (3.6)	5 (3.0)	
Antepartum complications during pregnancy, *n* (%)				0.44
Yes	94 (32.4)	20 (32.8)	74 (32.3)	
No	92 (31.7)	24 (39.3)	68 (29.7)	
Missing, *n* (%)	104 (35.9)	17 (27.9)	87 (38.0)	

*ART* assisted reproductive technology, *cm* centimeter, *g* grams, *IVF* in vitro fertilization, *kg* kilograms, *mm* millimeters, *NIFT* non-IVF fertility treatment, *SD* standard deviation, *UA* unassisted

^a^*p*-value from the Wilcoxon rank-sum test for continuous variables; Pearson’s chi-squared test for categorical variables; and Fisher’s exact test for sparsely distributed categorical data

Children from the UA + NIFT group were significantly older at baseline assessment compared to children in the ART group with mean ages of 8.2 ± 3.5 years (*n* = 61) and 7.2 ± 3.5 years (*n* = 229), respectively (*p* = 0.02). We did not observe differences between the ART and UA + NIFT groups in terms of race or ethnicity (*p* = 0.37 and *p* = 0.80, respectively). We did not observe any differences between the two comparison groups in terms of current household income (*p* = 0.38) or mother’s highest education (*p* > 0.9) but information was only available for about two-thirds of participants for these variables.

**Fig. 1:**
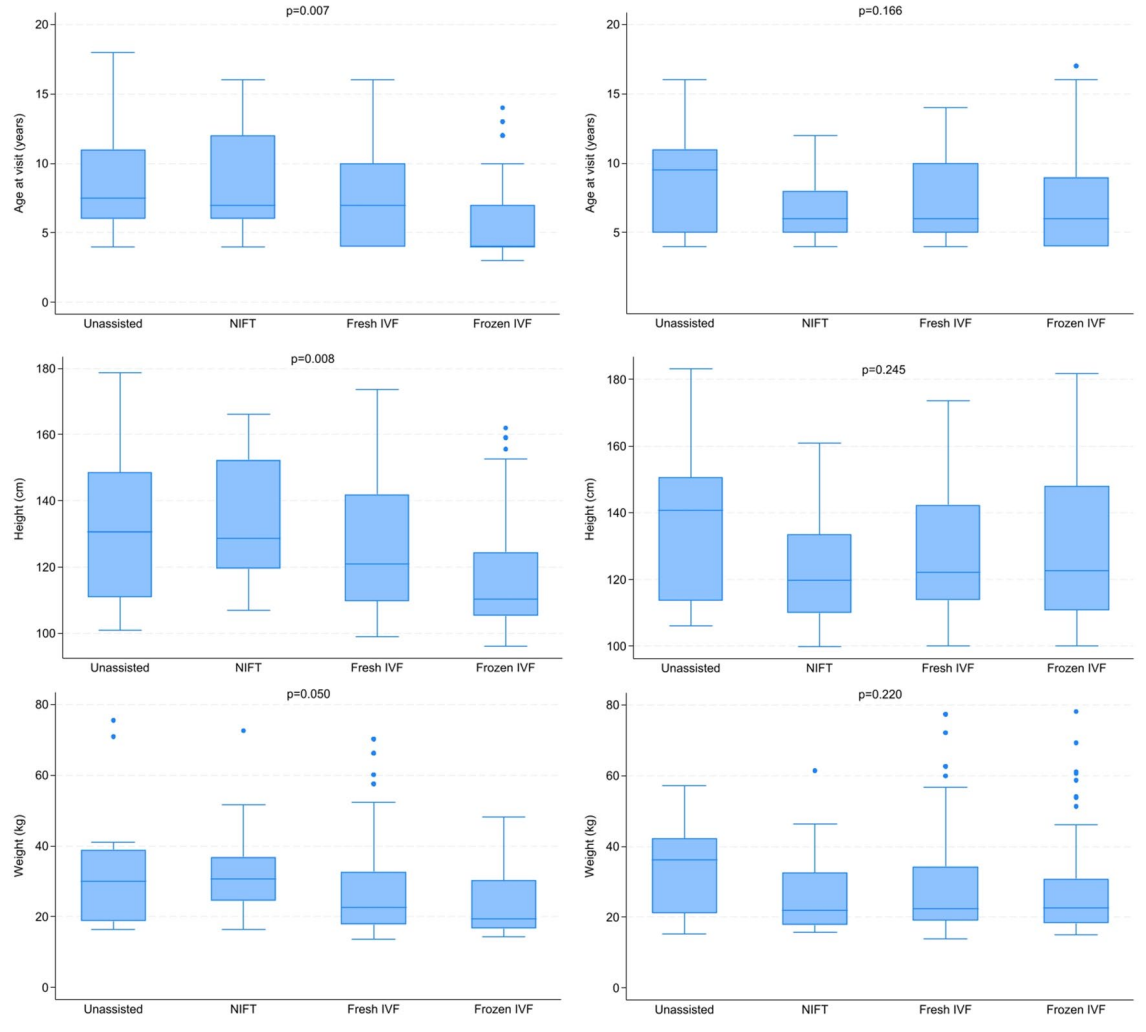
Characteristics of children by method of conception, separately for boys and girls, DESCRT study, 2017–2022. *Left*, characteristics of girls; *right*, characteristics of boys. IVF, in vitro fertilization; NIFT, non-IVF fertility treatment. *p*-values are derived from the Kruskal–Wallis test

The overall mean weight and height of the study population were 29 ± 14 kg and 128 ± 21 cm, respectively. UA + NIFT children had both higher mean height and mean weight compared to ART children (*p* = 0.04 and *p* = 0.02, respectively). We evaluated differences in age, height, and weight by method of conception for girls and boys (Fig. 1). We observed no differences in height and weight between girls and boys within categories of age (4–10 and 11–18 years); the only statistically significant difference was seen in girls aged 4 to 10 years, wherein the UA + NIFT group had a higher mean weight (*p* = 0.04).

Children from the ART group tended to have smaller mean tricep skinfold thickness compared to the UA + NIFT group but the difference was not statistically significant (*p* = 0.13), which may be related to younger gestational age at delivery and/or older maternal age at delivery observed for this group (*p* = 0.04 and *p* < 0.001, respectively). We did not observe any differences between the two comparison groups in terms of antepartum complications during pregnancy or birthweight (Table 1).

**Fig. 2:**
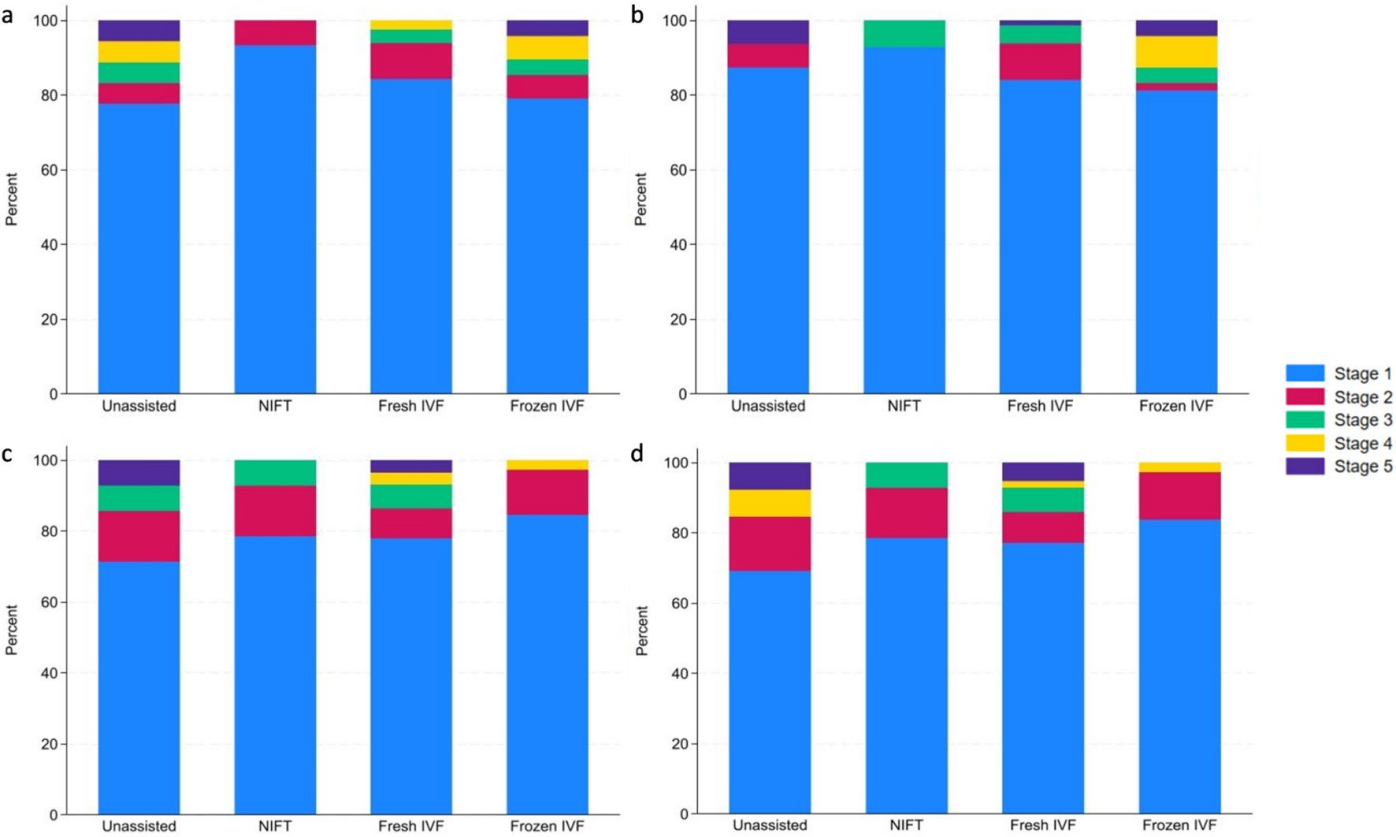
Percentages of boys and girls in Tanner stages 1–5, distributed by method of conception, DESCRT study, 2017–2022. **a** Pubarche stages in boys; **b** gonadarche stages in boys; **c** pubarche stages in girls; **d** thelarche stages in girls. IVF, in vitro fertilization; NIFT, non-IVF fertility treatment

Figure 2 illustrates the percentages of girls and boys in each Tanner stage distributed by method of conception. Five girls were missing a Tanner score for thelarche and four boys were missing a Tanner score for gonadarche. One hundred thirty-five (84.4%) boys were classified as being in stage 1 in terms of testicular development, and 95 (78.5%) girls received a breast Tanner score of 1. Of the children in the UA + NIFT group, 28 of 33 (84.8%) boys were classified as being in Tanner stage 1 for pubarche, while 21 of 28 (77.8%) girls were in Tanner stage 1 for pubarche. By comparison, in the ART group, we observed 108 of 131 (82.4%) boys and 79 of 98 (80.6%) girls in Tanner stage 1 for pubarche.

In total, 62 (21.4%) children were from a multiple birth, including 39 (23.8%) boys and 23 (18.3%) girls; multiple birth data were unreported for eight (2.7%) children. Of these multiple birth children, 59 (95.2%) were conceived via fresh IVF or frozen IVF (37 boys and 22 girls), versus three (4.8%) children conceived without assistance or IUI (two boys and one girl (*p* < 0.001)). The overall median age of children, excluding those born in a multiple birth, was 6 years (range, 4 to 18 years). After excluding multiple birth children, we still observed no difference in birthweight between comparison groups, and additionally found no significant differences in gestational age at delivery (*p* = 0.75 and *p* > 0.9, respectively).

### Comparison of Tanner stage by method of conception

After adjustment for children’s age, height, and weight, boys from the ART group had a higher odds of being in a later Tanner stage compared to the reference group for both pubarche (OR = 2.33, 95% CI 0.44–12.21) and gonadarche (OR = 4.27, 95% CI 0.44–41.99), although the increase was not statistically significant (*p* = 0.32 and *p* = 0.21, respectively). In girls, the ART group had higher odds of being in both later pubarche (OR = 4.29, 95% CI 0.40–45.62) and thelarche (OR = 2.23, 95% CI 0.35–14.03) stages but these increases similarly did not reach statistical significance (*p* = 0.23 and *p* = 0.39, respectively).

When children belonging to a multiple birth were excluded from analyses, boys in the ART group had a higher odds of being in a later pubarche Tanner stage than boys in the UA + NIFT group (OR = 1.53, 95% CI 0.23–10.12), but a lower odds of being in a later stage for gonadarche (OR = 0.88, 95% CI 0.03–23.54), although neither of these differences achieved statistical significance (*p* = 0.66 and *p* > 0.90, respectively). Girls in the ART group had higher odds of being in later Tanner stages for both pubarche (OR = 2.67, 95% CI 0.19–38.04) and thelarche (OR = 4.55, 95% CI 0.45–46.20), but these increases were not statistically significant (*p* = 0.47 and *p* = 0.20, respectively).

### Comparison of Tanner stage by fresh versus frozen embryo transfer

Because only seven girls from the UA + NIFT group were classified as being of a Tanner stage of 2 or higher, we conducted a sub-analysis of children conceived only via ART by method of embryo transfer (comparing fresh vs. frozen IVF). Of the boys conceived via fresh embryo transfer, 13 (15.7%) received a Tanner score of 2 or greater for pubarche, while 13 (15.9%) were scored 2 or greater for gonadarche. Ten (20.8%) boys conceived via frozen embryo transfer were scored 2 or greater for pubarche and 9 (18.8%) for gonadarche. Regarding girls conceived via fresh embryo transfer, 13 (22.0%) and 13 (22.8%) were scored 2 or greater for pubarche and thelarche, respectively. Six (15.4%) girls conceived via frozen embryo transfer were scored 2 or greater for pubarche and 6 (16.2%) for thelarche. After adjusting for children’s age, height, and weight, boys conceived via frozen embryo transfer had a higher odds of being in a later Tanner stage for pubarche (OR = 2.06, 95% CI 0.26–16.57) but not gonadarche (OR = 1.03, 95% CI 0.09–12.29), compared to those conceived via fresh embryo transfer, though neither association was statistically significant (*p* = 0.49 and *p* > 0.90, respectively). Girls conceived via frozen embryo transfer had lower odds of being in a later Tanner stage for thelarche compared to girls conceived via fresh transfer (OR = 0.76, 95% CI 0.07–8.72), but the trend was not statistically significant (*p* = 0.82). An analysis could not be performed on pubarche Tanner scores in girls due to insufficient statistical power. The results of the logistic regression analyses are summarized in Table 2.

**Table 2:** Estimates of odds ratios and 95% confidence intervals by Tanner stage separately for boys and girls, DESCRT study (2017–2022)

Exposure	Outcome	Boys					Girls				
			Crude		Adjusted^a^			Crude		Adjusted^b^	
		*N* (case/control)	OR	95% CI	OR	95% CI	*N* (case/control)	OR	95% CI	OR	95% CI
ART vs. UA + NIFT (reference)	Pubarche^c^	131/33	1.19	0.42–3.42	2.33	0.44–12.21	98/28	0.72	0.27–1.94	4.29	0.40–45.62
	Gonadarche^d^	130/30	1.83	0.51–6.58	4.27	0.44–41.99	-	-	-	-	-
	Thelarche^e^	-	-	-	-	-	94/27	0.72	0.27–1.96	2.23	0.35–14.03
Frozen IVF vs. fresh IVF (reference)	Pubarche	48/83	1.42	0.57–3.53	2.06	0.26–16.57	39/59	0.64	0.22–1.87	N/E	N/E
	Gonadarche	48/82	1.22	0.48–3.12	1.03	0.09–12.29	-	-	-	-	-
	Thelarche	-	-	-	-	-	37/57	0.66	0.22–1.91	0.76	0.07–8.72

*ART* assisted reproductive technology, *CI* confidence interval, *IVF* in vitro fertilization, *N/E* not estimated, *NIFT* non-IVF fertility treatment, *OR* odds ratio, *UA* unassisted

^a^Adjusted for child age (years), child height (cm), and child weight (kg)

^b^A small sample size led to overfitting (perfect prediction) of logistic regression models in some cases for girls; thus, these odds ratios are not reported

^c^Pubarche: pubic hair development (applicable to both girls and boys)

^d^Gonadarche: testicular development (only applicable to boys)

^e^Thelarche: breast development (budding; only applicable to girls)

After excluding children belonging to a multiple birth, boys conceived via frozen transfer had a higher odds of being later in pubarche Tanner stage compared to the fresh transfer group (OR = 1.74, 95% CI 0.14–22.18) but the difference was not statistically significant (*p* = 0.67). Girls conceived via frozen embryo transfer had a higher odds of being in a later thelarche stage compared to girls conceived via fresh transfer (OR = 2.17, 95% CI 0.06–79.76), but the difference similarly failed to reach statistical significance (*p* = 0.67). Insufficient statistical power prevented the analyses of gonadarche Tanner stage in boys and pubarche Tanner stage in girls. The results of the logistic regression analyses excluding children born in a multiple birth are presented in Table 3.

**Table 3:** Estimates of odds ratios and 95% confidence intervals by Tanner stage separately for boys and girls excluding children belonging to a multiple birth, DESCRT study (2017–2022)

Exposure	Outcome	Boys					Girls				
			Crude		Adjusted^a^			Crude		Adjusted^b^	
		*N* (case/control)	OR	95% CI	OR	95% CI	*N* (case/control)	OR	95% CI	OR	95% CI
ART vs. UA + NIFT (reference)	Pubarche^c^	91/28	0.84	0.27–2.57	1.53	0.23–10.12	74/27	0.68	0.23–2.03	2.67	0.19–38.04
	Gonadarche^d^	90/25	1.02	0.26–3.98	0.88	0.03–23.54	-	-	-	-	-
	Thelarche^e^	-	-	-	-	-	72/26	0.80	0.27–2.38	4.55	0.45–46.20
Frozen IVF vs. fresh IVF (reference)	Pubarche	36/55	0.82	0.25–2.69	1.74	0.14–22.18	33/41	0.36	0.09–1.44	N/E	N/E
	Gonadarche	36/54	0.84	0.23–3.10	N/E	N/E	-	-	-	-	-
	Thelarche	-	-	-	-	-	32/40	0.43	0.12–1.52	2.17	0.06–79.76

*ART* assisted reproductive technology, *CI* confidence interval, *IVF* in vitro fertilization, *N/E* not estimated, *NIFT* non-IVF fertility treatment, *OR* odds ratio, *UA* unassisted

^a^Adjusted for child age (years), child height (cm), and child weight (kg)

^b^A small sample size led to overfitting (perfect prediction) of logistic regression models in some cases; thus, these odds ratios are not reported

^c^Pubarche: pubic hair development (applicable to both girls and boys)

^d^Gonadarche: testicular development (only applicable to boys)

^e^Thelarche: breast development (budding; only applicable to girls)

## Discussion

In this cross-sectional analysis of baseline data on children from the DESCRT cohort study who were enrolled and evaluated for Tanner stage at a median age of 6 years, we found no statistical differences in pubertal development between ART and UA + NIFT groups but saw consistent indications of association between method of conception and Tanner stage attained. Boys and girls conceived via ART tended to be in later Tanner stages than those conceived via UA or NIFT. In addition, boys but not girls conceived via frozen embryo transfer tended to be in later Tanner stages compared to those conceived via fresh embryo transfer after adjustment for confounders, though the trends were not statistically significant. There also was a trend for more advanced Tanner stages among children conceived with IVF. Excluding children born in a multiple birth slightly altered the relationships observed, even reversing them in some cases, but these trends were similarly statistically non-significant. It is therefore possible that ART could affect the course of pubertal development in adolescent boys and girls, but further studies with larger sample sizes, and with broader distribution of ages, are needed to ascertain the exact nature of the association.

Our findings build on the existing literature, which present inconsistent results on the association between method of conception and pubertal presentation as assessed by Tanner stage [23–28]. Belva et al. previously reported girls conceived via ICSI had significantly less advanced breast development compared to girls conceived without assistance, as indicated in the present study (albeit non-significantly), with no difference between groups in terms of pubic hair development [24]. The same study reported no differences in pubertal development for boys. Conversely, Ernst et al. saw a tendency for girls born to subfecund parents to reach puberty milestones at earlier ages in both medically treated and untreated groups whereas a delay was seen in boys; neither trend was statistically significant [27]. In addition, Klemetti et al. concluded conception utilizing ART was significantly associated with both early and late puberty disorders, with early puberty more frequent in girls and late puberty more frequent in boys [30].

We attempted to include BMI-for-age, height-for-age, and weight-for-age and BMI *z*-scores in our logistic regression models, but controlling for these variables led to much lower predictive ability than controlling for age, height, and weight alone (results not shown). Several studies have examined the association between ART and body composition in adolescents since BMI and adiposity are associated with age of puberty onset, with contradictory results [16–19]. Ceelen et al. found increased in body fat in IVF-conceived children compared to UA controls when looking at skinfold measurements [45], while Meddeb et al. did not see a difference in the BMIs of children conceived via IVF/ICSI and UA up to 5 years of age [46], and Elhakeem et al. saw minimal differences in early life growth and adiposity in those conceived using ART [47]. While children in the unassisted conception group and NIFT group had significantly higher mean height and weight than children in the ART group, they were significantly older as well.

This study adds to the limited knowledge and inconsistent findings on the pubertal development of children conceived with ART compared to the UA and NIFT groups. Strengths of this study include a nearly complete ascertainment of the outcome variable and Tanner stage of enrolled children. Additionally, the DESCRT cohort is racially and ethnically diverse and representative of the source population of the San Francisco Bay Area and Northern California. However, we acknowledge that, given the relatively small size of the sample evaluated for Tanner stage in this study, the children who ultimately presented for the in-person examination may not have been representative. The young median age in our sample reflects opportunistic data collection: we assessed Tanner Stage in children aged 4 years and older from the DESCRT cohort who participated in in-person evaluations for adverse cardiometabolic outcomes. Furthermore, we faced the challenge of unreported/missing data on current household income, mother’s highest level of education attained, maternal age at delivery, and whether the mother experienced any complications or medical conditions during pregnancy, which may have limited our ability to adjust for known confounders or otherwise reduced statistical power of analyses.

## Conclusion

We found statistically non-significant differences in pubertal development between ART and UA + NIFT groups but observed consistent indications of association between method of conception and Tanner stage attained. Our study suggests a potential effect of ART, and specifically IVF, on advancing the timing of Tanner stages of children conceived with ART, but further research with larger sample sizes and statistical power are warranted. With the increased use of ART, there is urgency for future studies to examine long-term effects on pubertal development. Follow-up in children at older ages, including this cohort, is indicated.

## Data Availability

The dataset generated during and/or analyzed during the current study is available from the corresponding author on reasonable request.
